# Chemical composition and *in vitro* antibacterial activity of essential oil and methanol extract of *Echinophora platyloba D.C* against some of food-borne pathogenic bacteria

**Published:** 2013

**Authors:** Mohammad Hashemi, Ali Ehsani, Nima Hosseini Jazani, Javad Aliakbarlu, Razzaqh Mahmoudi

**Affiliations:** 1 *Department of Food Hygiene and Quality Control, Faculty of Veterinary Medicine, Urmia University, Urmia, Iran; *; 2 *Food and Beverages Safety Research Center, Urmia University of Medical Sciences, Urmia, Iran; *; 3 *Department of Food Hygiene and Aquatics, Faculty of Veterinary Medicine, University of Tabriz, Tabriz, Iran. *

**Keywords:** Antimicrobial activity, *Echinophora Platyloba D.C*, Essential oil, Methanol extract

## Abstract

*Echinophora Platyloba D.C *as a medicinal plant is used for preservation of foods and treatment of many diseases in different regions of Iran. The present study was undertaken to determine the chemical composition and investigation of the antibacterial effects of essential oil as well as methanol extract from aerial part of *Echinophora Platyloba D.C *against *S. aureus*,* L. monocytogenes*, *S. Thyphimurium* and *E. coli*. Chemical analysis using gas chromatography and mass spectrophotometry (GC/MS) showed that ocimene (26.51%), 2,3-Dimethyl-cyclohexa-1,3-diene (9.87%), alpha-pinene (7.69%) and gamma-dodecanolactone (5.66%) were dominant components of essential oil and the main constituents of methanol extract were o-Cymene (28.66%), methanol (8.50%), alpha-pinene (7.42%) and gamma-decalactone (5.20%). The essential oil showed strong antimicrobial activity against tested bacteria, whereas the methanol extract almost remained inactive against gram-negative bacteria. The most sensitive bacteria to essential oil and extract of *Echinophora Platyloba D.C *were *L. mono-cytogenes* and *S. aureus*. Minimum inhibitory concentration (MIC) values of essential oil against *L. monocytogenes* and *S. aureus* were 6250 and 12500 ppm, respectively. MIC of methanol extract against *S. aureus* and *L. monocytogenes* was 25000 ppm. Therefore, purifying and evaluation of antibacterial effects of the active substances of the essential oil and methanol extract of this plant for future application as antibacterial agents and food preservatives to combat pathogenic and toxigenic microorganisms is recommended.

## Introduction

Due to the side effects of chemical and synthetic antimicrobial agents and emerging increase in bacterial resistances to current antibiotics and other antimicrobial agents, more studies have been recently focused on characterization of novel potential natural antimicrobial agents from plant, animal and microbial sources.^1^ Plants are rich in a wide variety of secondary metabolites, such as tannins, terpenoids, alkaloids and flavonoids, which have been reported to have *in vitro *antimicrobial properties.^2^ Essential oils and their components are gaining increasing interest because of their relatively safe status, their wide acceptance by consumers and their exploitation for potential multi-purpose functional use.^3^ Many researchers have reported the antimicrobial, antifungal and anti-oxidant properties of essential oils.^4^^-^^8^

 A knowledge of the chemical constituents of plants is desirable not only for the discovery of therapeutic agents but because such information may be of value in disclosing new sources of such economic materials as tannins, oils, gums, precursors for the synthesis of complex chemical substances, etc. In addition, the knowledge of the chemical constituents of plants would further be valuable in discovering the actual value of folkloric remedies.^5^ The genus *Echinophora* (*Umbelliferae*, subfamily *Apioideae,* tribe *Echinophoreae*), is represented in the plant flora of Iran.^9^ *Echinophora Platyloba D.C*, a member of the *Umbelliferae* family, is a perennial plant, distributed only in Mediterranean region mostly at maritime sands also which could be found in some central and western provinces of Iran.^6^^,^^10^ The underground rhizome has a wide growth and the erected stem is full of branches. Leaves end with spines. It blossoms from June to September. In Iran, fresh and dried aerial parts of some of these species are added to cheese and yoghurt for flavoring, the species of the *Echinophora *genera are also used in folk medicine to heal wounds and to treat gastric ulcers due to its anti-fungal, carminative, and digestive properties,^11^ as a stimulant and an invigorator of the stomach and its anti-microbial and anti-cancer effects have been shown, recently.^12^

The objectives of this study were 1) to determine the chemical composition (based on GC-MS results) of hydro-distilled essential oils and methanol extract of *Echinophora Platyloba D.C *and 2) to investigate their antibacterial activities.

## Materials and Methods

**Plant material. **The aerial parts of *Echinophora Platyloba D.C *was collected during flowering stage (10^th^ June to 15^th^ August 2010) from Maragheh city, northwest of Iran and identified by the Herbarium of West Azerbaijan Agricultural and Natural Resource Center, Urmia, Iran (Voucher specimen no. : 6502). Then, it was dried and ground into powder. The prepared powder was kept in tight containers protected completely from light.

**Preparation** **of the essential oil. **Dry aerial parts (100 g) of *Echinophora Platyloba D.C *were subjected to the hydrodistillation for 2.5 hr, using a clevenger-type apparatus, according to the method recommended by the European Pharmacopia.^13^^,^^14^ The obtained essential oil was dried over anhydrous sodium sulphate and stored at 4 ˚C for further experiments.

**Preparation of methanol extract. **The air-dried and finely ground sample was extracted using the method described previously.^15^ Briefly, 100 g of sample were extracted in a Soxhlet with methanol in 60 ˚C for 6 hr. The extracts were then filtered with Whatman filter paper number 1 and concentrated *in vacuo *at 45 ˚C by a rotary evaporator (Heidolph laborta 4003, Schwabach, Germany) yielding a waxy material. Extracts were concentrated *in vacuo*, dried, and kept in the dark at 4 ˚C until tested.

**Analysis of the essential oil and extract. **Chemical composition of the essential oil and extract were analyzed by gas chromatography. The gas chromatograph (Agilent 6890, Swindon, UK) was equipped with an HP-5MS capillary column (30 × 0.25 mm ID × 0.25 mm film thickness) and the data were taken under the following conditions: initial temperature 50 ^°^C, temperature ramp 5 ^°^C per min, 240 ^°^C min to 300 ^°^C (holding for 3 min), and injector temperature at 290 ^°^C. The carrier gas was helium and the split ratio was 0.8 mL^-1^ per min. For confirmation of results, essential oil was also analyzed by gas chromato-graphy mass spectrometry (Agilent 6890 gas chromatograph equipped with an Agilent 5973 mass-selective detector; Agilent, Swindon, UK) and the same capillary column and analytical conditions were used as mentioned above. The MS was run in electron-ionization mode with ionization energy of 70 eV (Wiley-VCH 2001, Weinheim, Germany).

**Bacterial strains. **The essential oil and extract were individually tested against two gram-negative (*S. Thyphimurium* ATCC 13311 and *E. coli* ATCC 43894) and two gram-positive bacteria (*S. aureus* ATCC 6538,* L. monocytogenes* ATCC 19118). Lyophilized cultures of the organisms were obtained from the culture collection of the Department of Microbiology, Faculty of Veterinary Medicine, University of Tehran, Tehran, Iran.

**Micro-well dilution assay. **The minimum inhibitory concentration (MIC) and minimum bactericidal concentration (MBC) values of the essential oil and extract were studied for the bacterial strains in micro plates. The inocula of the bacterial strains were prepared from 18 hr nutrient broth cultures and suspensions were adjusted to 0.5 McFarland standard turbidity. Essential oil and extract were dissolved in 10% dimethyl sulfoxide. Then, the solution firstly was diluted to the highest concentration (100000 ppm) as a stock solution, and then serial two-fold dilutions were made in a concentration range from 1562.5 to 100000 ppm in nutrient broth. Minimum inhibitory concentration values of essential oil and extract against bacterial strains were determined based on a micro-well dilution method. The 96-well plates were prepared dispensing 160 µL of nutrient broth and 20 µL of the inoculums into each well. A 20 µL aliquot from the stock solutions of essential oil initially prepared at the concentration of 100000 ppm were added into the first wells. Then, 20 µL from their serial dilutions was transferred into consecutive wells. The last well containing 180 µL of nutrient broth without any chemical compound and 20 µL of the inoculum on each strip which were used as the negative control. The final volume in each well was 200 µL.^16^ The plates were covered with a sterile plate sealer. Contents of each well were mixed on plate shaker at 300 rpm for 20 sec and then incubated at appropriate temperatures for 24 hr. Microbial growth was determined by absorbance at 600 nm using the EL_x_ 800 universal micro-plate reader (Biotek Instrument Inc., Winooski, VT, USA) and confirmed by plating 5 µL samples from clear wells on nutrient agar medium. The MIC and MBC were defined as the lowest concentration of the compounds to inhibit the growth of microorganisms and show bactericidal effects on micro-organisms, respectively.^17^^,^^18^

**Statistical analysis. **All data were expressed as mean ± standard deviations (SD) of triplicate measure-ments. Data analysis was performed using Graphpad Prism (Version 5.0 for Windows, Graphpad software, San Diego, CA, USA). 

## Results

The yield of essential oil and methanol extract were 0.3% and 1.5% (v/_W_) based on dry weight, respectively. The chemical compositions of *E. platyloba D.C* essential oil and extract are summarized in Tables 1 and 2. Ninety and 21 compounds were characterized representing 85.42% and 91.68% of the total content of essential oil and methanol extract, respectively. 

The major compound of essential oil was ocimene (26.51%), following by 2,3 Dimethyl-1,3-cyclohexadiene (9.87%), alphapinene (7.96%), gamma-dodecalactone (5.84%) and nerolidol (5.66%). Ocimene (28.66%) was the most abundant components in *E. platyloba D.C *methanol extract and the other major components were beta-cis-ocimene (9.77%), methanol (8.57%), alpha-pinene (7.42%), gamma.-decalactone (5.20%) and beta-linalool (4.99%). The minimum inhibitory concentration and MBC values of* E. platyloba D.C* essential oil and extract on different kinds of bacteria in this study are summarized in Tables 3 and 4. The essential oil showed strong antimicrobial activity against tested bacteria, whereas the methanol extract almost remained inactive against gram-negative bacteria. The most sensitive bacteria to essential oil and extract of *Echinophora Platyloba D.C *were *L. monocytogenes* and *Staphylococcus aurues*, MIC values of essential oil against *L. monocytogenes* and *S. aureus* were 6250 and 12500 ppm, respectively. The minimum inhibitory concentration of extract against *S. aureus* and *L. monocytogenes* was 25000 ppm.

**Table 1:** Chemical composition (%) of *Echinophora Platyloba D.C* essential oil analyzed by GC/MS.

**Compound**	**KI**	**Composition**
Hexanal	806	1.25
2,3-Dimethyl-cyclohexa-1,3-diene	863	9.87
Alpha-pinene	948	7.69
Ocimene	958	26.51
Beta-linalool	1082	1.80
Benzopyran	1342	1.18
Cyclohexene, 2-ethenyl-1,3,3-trimethyl	1105	1.44
2,5-Octadecadiynoic acid, methyl ester	2112	2.30
Caryophyllene	1494	2.48
Dihydropseudoionone	1420	1.48
Gamma-dodecanolactone	1582	5.84
4-(2,2-Dimethyl-6-methylenecyclohexylidene) -3-methylbutan-2-1	1475	1.13
Nerolidol	1564	5.66
All-trans-farnesol	1710	3.30
Gamma-dodecalactone	1582	3.28
Heptacosane	2705	2.10
Nonacosane	2904	4.11
Cis-Z-alpha-bisabolene epoxide	1531	1.11
2-[4-6-(2,6,6-trimethylcyclohex-1-enyl)hexa-1,3,5-trienyl]cyclohex-1-en-1-carboxaldehyde	2561	2.89
**Sum**	-	85.42

**Table 2:** Chemical composition (%) of *Echinophora Platyloba D.C* methanolic extract analyzed by GC/MS.

**Compound**	**KI**	**Composition**
Methanol	315	8.50
Alpha-pinene	948	7.42
Beta-terpinene	1071	1.49
Beta-myrcene	993	3.22
O-cymene	1028	28.66
beta.-cis-Ocimene	1040	9.77
Isopinocarveol	1198	1.06
Beta-Linalool	1109	4.99
13-Tetradece-11-yn-1-ol	1663	1.08
2-Nonenal	1161	1.31
Naphthalene	1157	3.47
5-Isopropenyl-2-methyl-7-oxabicyclo[4.1.0]-heptan-2-ol	1294	1.42
Acetic acid	1009	1.84
Trans-Z-alpha-bisabolene epoxide	1746	1.34
5,6,6-Trimethyl-5-(3-oxobut-1-enyl)-1-oxaspiro[2.5]octan-4-1	1442	1.00
Gamma-decalactone	1431	5.20
Formic acid	1576	1.24
Spathulenol	1699	2.92
Trans-Farnesol	2021	3.17
2-Butyloxycarbonyloxy-1,1,10-trimethyl-6,9-epidioxydecalin	3942	1.60
1.3-Ethyl-5-(2'-ethylbutyl)octadecane	3430	1.00
**Sum**	-	91.68

**Table 3:** Antimicrobial properties (MIC and MBC) of *Echinophora Platyloba D.C* essential oil.

**Microorganisms**	**MIC (ppm)**	**MBC (ppm)**
***S. aureus***	12500	25000
***L. monocytogenes***	6250	6250
***E. coli***	50000	
***S. typhimurium***		

* No effect was determined while the highest concentration was used.

**Table 4:** Antimicrobial properties (MIC, MBC) of *Echinophora Platyloba D.C* methanolic extract.

**Microorganisms**	**MIC (ppm)**	**MBC (ppm)**
***S. aureus***	25000	
***L. monocytogenes***	25000	
***E. coli***		
***S. typhimurium***		

* No effect was determined while the highest concentration was used.

## Discussion

The oil composition of different species from *Echinophora *genus has been studied and various compounds such as 3-carene,^9^ (E)-β-ocimene,^19^ a-phellandrene^20^ and delta-3-carene^21^ have been reported as the first major constituent. Rahimi-Nasrabadi *et al*. indicated that major essential oil composition of the aerial parts of *Echinophora platyloba *growing wild in Isfahan province of Iran is bata-ocimene, delta-3-carene and limonene which are quite consistent with the results of chemical analysis of current study.^19^

Most studies concerning the antimicrobial mode of action of essential oil constituents have been performed on bacteria, gram-negative bacteria are generally less susceptible than gram-positive bacteria.^22^ The outer membrane of gram-negative bacteria contains hydrophilic lipopolysaccharides (LPS), which create a barrier toward macromolecules and hydrophobic compounds, providing gram-negative bacteria with higher tolerance toward hydrophobic antimicrobial compounds like those found in essential oils.^23^ Minimum inhibitory concentration and MBC values of* E. platyloba D.C* essential oil on different kinds of bacteria in this study indicated the notable sensitivity of gram-positive bacteria and the relative susceptibility of gram-negative bacteria.

An study by Entezari *et al*. indicated that methanolic extract of *E. platyloba D.C* can inhibit the growth of two bacterial species of *S. aureus* and *P. aeruginosa*.^3^ In the current study *S. aureus* was one of the most sensitive tested bacteria to essential oil and extract of *Echinophora Platyloba D.C *as well and it’s growth was inhibited in MIC values of 12500 and 25000 ppm for essential oil and extract, respectively.

Results of this study suggested that *E. platyloba D.C* essential oil and extract have potential effects as antimicrobial agents. Main components of essential oil and extract, such as ocimene, α-pinene, myrcene and α-phellandrene have been previously reported to have antibacterial activity.^11^

The carvacrol precursor p-cymene is a monoterpene that has a benzene ring without any functional groups on its side chains. P-cymene has a high affinity for membranes and causes membrane expansion and affect the membrane potential of intact cells.^24^ P-cymene had a negligible effect on the protein synthesis of *E. coli *cells, while its effect on the membrane potential resulted in decreased cell motility, as a proton motive force is needed for flagellar movement.^25^

Terpenes are hydrocarbons produced from combination of several isopreneunits (C5H8). In a large scale experiment, limonene, α-pinene, β-pinene, δ-3-carene, sabinene, and α-terpinene showed no or low antimicrobial activity against 25 different genera of bacteria that pose problems in animals, plants, and food products.^26^ Koutsoudaki et al. compared the antibacterial effects of α-pinene, β-pinene, p-cymene, β-myrcene, β-caryophyllene, limonene and γ-terpinene on *E. coli*, *S. aureus* and *Bacillus cereus*, their antimicrobial activity were low or absent.^27^ The p-cymene and γ-terpinene were ineffective as fungicides against *Saccharomyces cerevisiae*.^28^ These *in vitro *tests indicate that terpenes are inefficient as antimicrobials when applied as single compounds.

In conclusion, regarding this study, it is clear that *E. platyloba D.C* essential oil and its methanol extract indeed exhibit antibacterial activity. Its antibacterial activity against *L. monocytogenes *was the highest followed by *S. aureus *and *E. coli*. Therefore, it can be suggested to purify and evaluate the antibacterial effects of active substances of *E. platyloba D.C* essential oil and methanol extract for therapeutic or industrial utilization.
